# A Novel Flow Cytometry-Based Assay for the Identification of HCN4 CNBD Ligands

**DOI:** 10.3390/ph16050710

**Published:** 2023-05-07

**Authors:** Magdalena N. Wojciechowski, Sebastian Schreiber, Joachim Jose

**Affiliations:** University of Münster, Institute of Pharmaceutical and Medicinal Chemistry, Pharmacampus, 48149 Münster, Germany

**Keywords:** autodisplay, HCN channels, CNBD, ligand binding

## Abstract

Hyperpolarization-activated and cyclic nucleotide-gated (HCN) channels are promising therapeutic targets because of their association with the genesis of several diseases. The identification of selective compounds that alter cAMP-induced ion channel modulation by binding to the cyclic nucleotide-binding domain (CNBD) will facilitate HCN channel-specific drug development. In this study, a fast and protein purification-free ligand-binding approach with a surface-displayed HCN4 C-Linker-CNBD on *E. coli* is presented. 8-Fluo-cAMP ligand binding was monitored by single-cell analysis via flow cytometry, and a K_d_-value of 173 ± 46 nM was determined. The K_d_ value was confirmed by ligand depletion analysis and equilibrium state measurements. Applying increasing concentrations of cAMP led to a concentration-dependent decrease in fluorescence intensity, indicating a displacement of 8-Fluo-cAMP. A K_i_-value of 8.5 ± 2 µM was determined. The linear relationship of IC_50_ values obtained for cAMP as a function of ligand concentration confirmed the competitive binding mode: IC_50_: 13 ± 2 µM/16 ± 3 µM/23 ± 1 µM/27 ± 1 µM for 50 nM/150 nM/250 nM/500 nM 8-Fluo-cAMP. A similar competitive mode of binding was confirmed for 7-CH-cAMP, and an IC_50_ value of 230 ± 41 nM and a K_i_ of 159 ± 29 nM were determined. Two established drugs were tested in the assay. Ivabradine, an approved HCN channel pore blocker and gabapentin, is known to bind to HCN4 channels in preference to other isoforms with an unknown mode of action. As expected, ivabradine had no impact on ligand binding. In addition, gabapentin had no influence on 8-Fluo-cAMP’s binding to HCN4-CNBD. This is the first indication that gabapentin is not interacting with this part of the HCN4 channel. The ligand-binding assay as described can be used to determine binding constants for ligands such as cAMP and derivatives. It could also be applied for the identification of new ligands binding to the HCN4-CNBD.

## 1. Introduction

**Figure 1:**
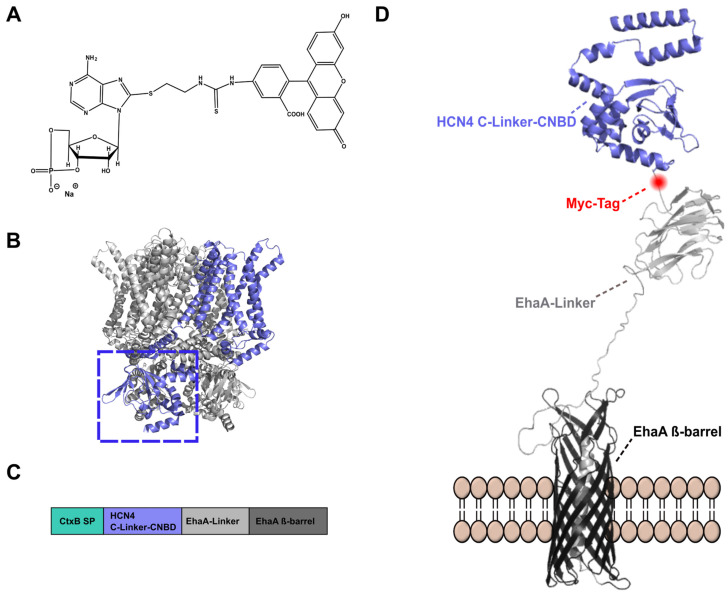
Graphical representation of surface-displayed HCN4 C-Linker-CNBD. (**A**) Structure of 8-Fluo-cAMP. (**B**) HCN4 tetrameric crystal structure (PDB:6GYN), one monomer (blue) consists of an N-terminal domain, transmembrane domains and an intracellular C-Linker-CNBD domain (framed). (**C**) Schematic representation of the domains required for autodisplay: CtxB signal peptide (SP), passenger domain consisting of HCN4 C-Linker-CNBD and the translocator domain consisting of EhaA-Linker and EhaA ß-barrel. (**D**) HCN4 C-Linker-CNBD (blue; PDB: 3OTF) presented on the surface of *E. coli* by the EhaA translocator domain consisting of an EhaA ß-barrel (black) and the EhaA-Linker (silver) with a Myc-Tag (red), provided by D. Gercke [44].

Hyperpolarization-activated and cyclic nucleotide-gated (HCN) channels belong to the superfamily of voltage-gated pore loop channels such as CNG channels and EAG-like K^+^ channels [1]. Upon hyperpolarization, a mixed Na^+^/K^+^ inward pacemaker ion current I_f_ (funny) is generated in the heart region and an I_h_ (hyperpolarization-activated) current is generated in the brain region [2]. HCN channels are encoded by four genes (HCN1-4) [3,4] and homo- or heterotetrametric proteins were formed within the membrane [5]. Crystal structures were obtained for HCN1 [6,7] and HCN4 [8] and it was revealed that each monomer consists of an N-terminal intracellular HCN-domain, followed by six α-helical transmembrane segments (S1–6). S1–4 constitute the voltage–sensor–domain (VSD) and S5–6 form the pore domain that includes the selectivity filter. The C-terminus is located intracellularly and comprises the cyclic nucleotide-binding domain (CNBD), which is connected to S6 by the C-Linker. Endogenous cyclic nucleotides, such as cAMP or cGMP, induce a voltage shift to more depolarized potentials upon binding to the CNBD and alter channel opening and closing kinetics. The extent of cyclic nucleotide modulation differs between the HCN subtypes [9,10,11]. Expression patterns of the isoforms are varying in the brain, heart, retina and peripheral nervous system [12,13,14]. HCN channels are involved in the regulation of cardiac and neuronal rhythmicity, generation of the resting membrane potential, dendritic integration or synaptic transmission [15]. Therefore, it appears evident that HCN channelopathies contribute to the pathogenesis of several diseases, including bradycardia [16], arrhythmia [17], epilepsy [18,19] or neuropathic pain disorder [20,21]. Although HCN channels were discovered more than 40 years ago [22,23,24] and their involvement in different diseases has been described in many studies [25,26], only a single drug has been approved until now, addressing HCN channels as targets. Ivabradine, a non-selective HCN channel pore blocker, was approved for the treatment of stable angina pectoris and heart failure disease [27]. Identification of new drugs addressing HCN channel isoforms selectively could be a promising option for novel specific therapies. At least two ways are possible by which a compound can target a HCN channel: either by blocking the pore like ivabradine or by interacting with the CNBD, and hence altering the cAMP-induced channel modulation. So far, drug discovery approaches have solely identified compounds targeting the HCN channel pore. By the remaining 7,8-dimethoxy-benzazepinone moiety and modification of the propyl chain of the non-selective pore blocker Zatebradine, subtype-selective HCN channel pore blockers as MEL57A, MEL55A and EC18 were developed [28,29,30]. Nakashima et al. (2021) identified novel HCN4 channel pore blockers, T-478, a methoxybenzenesulfonamide derivate, T-788, a tetrahydrooxazolopyrazinone derivate, and T-524, a thetrahydroisoquinoline derivate. However, these compounds were tested exclusively on HCN4 blocking, and selectivity tests with other HCN isoforms are still lacking [31]. Several other drugs were described to modulate HCN channel activity in addition to primary targets. This includes general and local anesthetics, anticonvulsive drugs and plant-derived compounds [32,33,34]. The application of such drugs for the treatment of HCN channel-dependent diseases is limited due to non-selectivity and hence risks severe side effects. To date, no compounds targeting the CNBD of HCN channels selectively have been described [32]. The first comprehensive study on a range of cyclic nucleotide derivatives binding to the CNBD of HCN1, 2 and 4 provided structural information about moieties required for ligand binding [35]. Studies with HCN C-Linker-CNBD applied surface plasmon resonance (SPR) [36], isothermal titration calorimetry (ITC) [9] or fluorescence anisotropy (FA) [35] for measuring the binding to the corresponding C-Linker-CNBD. For these purposes, protein purification was necessary. Successful purification of recombinant proteins can be impaired by several parameters including low yield, solubility and protein-folding issues or proteolytic degradation [37,38]. A strategy to circumvent these problems is to tag a second protein, e.g. the maltose-binding protein (MBP), to the target protein [38,39]. In this study, a new autodisplay-based [40,41] ligand-binding assay for the HCN4 C-Linker-CNBD was developed, which avoids the need for protein purification and provides protein stability. For this purpose, the HCN4 C-Linker-CNBD (Figure 1B) was displayed on the surface of *E. coli*. Fluorescent cAMP derivative 8-Fluo-cAMP (Figure 1A) was used to determine ligand binding to the CNBD by single-cell analysis via flow cytometry [42,43]. The assay conditions were rectified with regard to equilibrium state conditions, ligand depletion and non-specific ligand binding. 

## 2. Results

### 2.1. Autodisplay of the HCN4 C-Linker-CNBD

Autodisplay is a technique that employs the natural type V autotransporter secretion pathway in Gram-negative bacteria to present proteins of interest on the bacterial surface [40]. The gene construct for surface display of HCN4 C-Linker-CNBD was based on the maximized autotransporter-mediated expression (MATE) system as described previously [45]. The artificial gene encoded a sequence for an N-terminal CtxB signal peptide, which is cleaved off after the translocation across the inner membrane, the passenger domain human HCN4 C-Linker-CNBD and the autotransporter translocator domain, consisting of an EhaA-Linker (including a Myc-tag) and the EhaA ß-barrel [45] (Figure 1C,D). Successful HCN4 C-Linker-CNBD surface display was confirmed via immunolabeling and flow cytometry (Figure S1). *E. coli* BL21 cells expressing the fusion protein were incubated with the primary anti-Myc-tag antibody and a secondary antibody conjugated to a DyLight^TM^ 633 fluorophore. *E. coli* BL21 cells without plasmid served as a control. Flow cytometer analysis indicated an increased mean fluorescence intensity (mFI) for HCN4 C-Linker-CNBD displaying cells when compared with control cells (Figure S1A). This was, on the one hand, to prove the surface display of the recombinant protein and on the other hand, it indicated the non-permeability of the fluorophore through the bacterial membrane. The incubation of such cells with Proteinase K leads to the digestion of surface-displayed proteins because the enzyme is not able to cross the bacterial outer membrane as has been shown by many examples before [46]. After the Proteinase K treatment, cells expressing the HCN4 C-Linker-CNBD fusion protein showed a decreased mFI compared with the same cells untreated (Figure S1B). This confirmed the surface display of the HCN4 C-Linker-CNBD. As a further control, cells displaying the target protein were treated with the secondary antibody only to exclude non-specific binding events. Flow cytometry analysis showed no increase in mFI for cells treated with the secondary antibody alone when compared to cells that were treated with the primary and secondary antibodies. This result confirmed the specific binding of the primary antibody (Figure S1C).

### 2.2. Binding of 8-Fluo-cAMP to Surface-Displayed HCN4 C-Linker-CNBD

**Figure 2:**
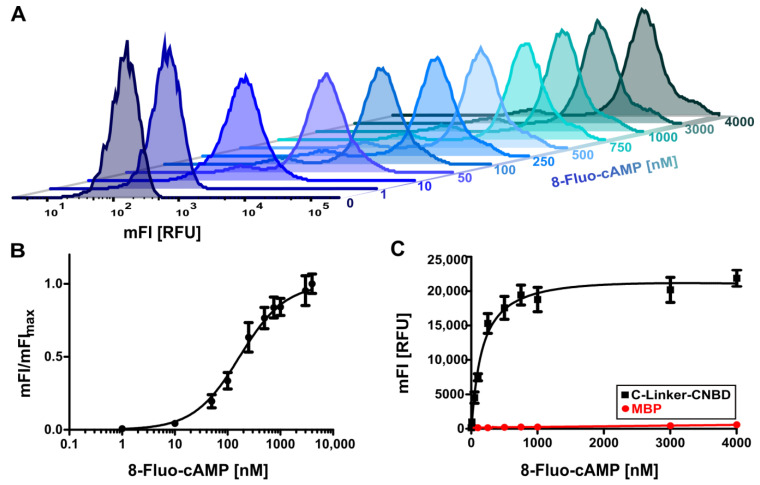
Flow cytometry-based ligand-binding assay with surface-displayed HCN4 C-Linker-CNBD and 8-Fluo-cAMP. (**A**) Flow cytometry histogram obtained for cells presenting the HCN4 C-Linker-CNBD after incubation with increasing concentrations of 8-Fluo-cAMP ranging from 1 nM–4 µM. (**B**) Ligand-binding curve of 8-Fluo-cAMP binding to cells presenting HCN4 C-Linker-CNBD. Mean fluorescence intensity (mFI) of each ligand concentration was normalized to the mFI of the highest ligand concentration used (mFI_max_) and plotted against the ligand concentration. (**C**) Comparison of 8-Fluo-cAMP ligand binding to cells displaying HCN4 C-Linker-CNBD (black) or maltose-binding protein (MBP) (red). Absolute mFI obtained for each sample is plotted against the ligand concentration used.

The intrinsic fluorescence of 8-Fluo-cAMP enabled the direct measurement of binding without additional labelling steps. Bacterial cells displaying the HCN4 C-Linker-CNBD were incubated with 8-Fluo-cAMP in concentrations ranging from 1 nM to 4 µM. Whole-cell fluorescence (mFI) monitored by flow cytometry indicated a concentration-dependent increase in linear relationship to the 8-Fluo-cAMP concentration (Figure 2A). Saturation appeared to be attained at a concentration of around 500 nM 8-Fluo-cAMP. Plotting the ligand fraction bound, derived from the mFI values against the ligand concentration, resulted in an ordinary binding curve (Figure 2B). The binding curve obtained was used to determine the dissociation constant (K_d_) of 8-Fluo-cAMP with the surface-displayed HCN4 C-Linker-CNBD and turned out to be 173 ± 46 nM. This was in good agreement with those binding affinities that have been reported before, as determined with fluorescence polarization (FP) ranging from 111 nM [47] to 189 nM [35], when bound to purified HCN4 MBP-C-Linker-CNBD fusion protein, and ranging from 167 nM [47] to 280 nM [9] when determined with purified HCN4 C-Linker-CNBD. 

### 2.3. Evaluation of Assay Conditions

To evaluate whether the conditions chosen were adequate, three scenarios were taken into account: unspecific ligand binding to the surface-displayed protein, non-equilibrium state conditions and a potential effect of ligand depletion. As a control for unspecific protein binding, surface-displayed MBP was used because 8-Fluo-cAMP is not supposed to have any affinity to MBP. When cells displaying MBP were treated with the same concentrations of 8-Fluo-cAMP as cells displaying HCN4 C-Linker-CNBD, only a marginal increase in mFI was observed (Figure 2C). In contrast, cells displaying the HCN4 C-Linker-CNBD showed a concentration-dependent increase in mFI with the saturation of binding attained at around 500 nM, as expected. This clearly indicated a specific binding of 8-Fluo-cAMP to the CNBD and a negligible unspecific protein binding. 

To elucidate whether the ligand-binding assay was performed under equilibrium state conditions, the incubation time of 8-Fluo-cAMP with HCN4 C-Linker-CNBD displaying cells was doubled from 30 min to 60 min. It was to be expected that in the case of non-equilibrium state conditions, the ligand-binding curve as obtained should be shifted to lower values after 60 min incubation time [48]. The binding curves obtained with the same concentrations of 8-Fluo-cAMP after 30 min and after 60 min appeared to be identical (Figure S2A). This indicated that an incubation time of 30 min is sufficient to obtain the equilibrium state of 8-Fluo-cAMP binding to CNBD. 

Generally, in ligand-binding studies, it is assumed that the total amount of ligand as applied is large enough to avoid the fact that the free ligand concentration is considerably altered upon ligand binding, i.e., the amount of bound ligand is, by order of magnitude, lower than the amount of ligand as added. Ligand depletion with a supposed impact on the result of the binding assay occurs when the free ligand concentration is substantially decreased upon ligand binding. To avoid ligand depletion conditions, the ratio of the total amount of ligand binding sites to the total amount of the ligand added to the system should not exceed 10% [48,49]. To investigate whether the amount of ligand as applied was sufficient to avoid ligand depletion, the reaction volume was increased from 100 µL to 200 µL, leaving the 8-Fluo-cAMP concentrations identical. The total number of cells presenting the target protein remained constant and the total amount of ligand added to the samples would need to be increased to obtain the same molar concentration in the 200 µL samples [48]. In the case of ligand depletion, the ligand-binding curve obtained for a 100 µL sample volume would be rightward shifted when compared to the binding curve obtained for a 200 µL sample volume. Flow cytometry analysis and the comparison of the binding curves obtained for samples with the same concentrations of 8-Fluo-cAMP in 100 µL and 200 µL reaction volumes showed no difference. This indicated that under the assay conditions as applied, ligand depletion appeared to not be of influence (Figure S2B).

To elucidate the affinity range of the assay, the amount of surface-displayed C-Linker-CNBD protein was determined by densitometry as described before by Tian et al., 2022 [50]. It was calculated to be 5.1 × 10^4^ molecules per cell, which was in the same order of magnitude as reported before for other surface-displayed proteins [40,50,51]. Since a minimum number of 10^5^ cells is required for a flow cytometry sample [48], a minimum number of approximately 10^9^–10^10^ receptors can be calculated per sample. The entire number of ligands as applied needs to exceed the maximum amount of bound ligand (equal to 10^9^–10^10^ receptors sites) by a factor of 10, as described above, the lower limit of K_d_ values as determinable with this assay appeared to be in the low nM range. The upper limit of the assay can be derived from the amount of unspecific bound 8-Fluo-cAMP to control cells. Unspecific binding to the cells depends on the ligand as such and has to be determined prior to K_d_ estimation. In the present study, no unspecific binding of 8-Fluo-cAMP to surface-displayed MBP (control) was observed up to 4 µM. Higher concentrations have not been applied. Hence, the affinity range of the assay is supposed to be in between the low nM and the medium or high µM range. 

The robustness of the assay was estimated by comparing the values as obtained for 8-Fluo-cAMP in measurements performed as biological replicates. Here, it needs to be taken into account that vivid bacterial cells were applied, which indeed could exhibit an intra-assay and an inter-assay variance, and hence, the absolute values for the mFI of 8-Fluo-cAMP bound to the CNBD can differ on different days. However, when comparing mFI values normalized by mFI_max_ of the same series of measurements, this resulted in almost identical mFI_norm_ values, binding curves and calculated binding affinities (data not shown). This indicated a high robustness of the assay as described here.

### 2.4. Binding of cAMP to Surface-Displayed HCN4 C-Linker-CNBD

**Figure 3:**
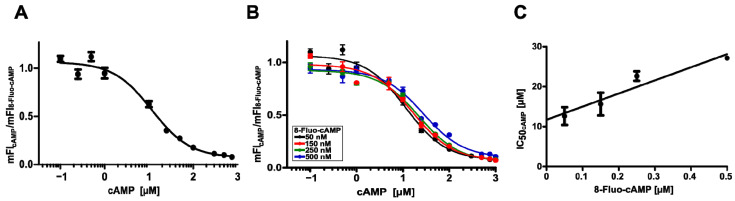
Competitive binding assay with 8-Fluo-cAMP and cAMP. (**A**) Displacement-binding curve obtained for cells displaying the HCN4 C-Linker-CNBD after incubation with 50 nM 8-Fluo-cAMP (ligand) and increasing concentrations of cAMP (competitor) ranging from 100 nM to 1 mM. (**B**) Ligand displacement-binding curves obtained at different ligand concentrations: 50 nM (black), 150 nM (red), 250 nM (green) or 500 nM (blue) 8-Fluo-cAMP. (**C**) Plot of the calculated IC_50_ values for cAMP against ligand concentration (R^2^ = 0.86).

To measure the binding of cAMP to surface-displayed HCN4 C-Linker-CNBD in a quantitative manner, corresponding cells were incubated with a fixed concentration of 8-Fluo-cAMP (50 nM) and increasing concentrations of cAMP reaching from 100 nM to 1 mM. This resulted in decreasing mFI values with increasing cAMP concentration as measured by flow cytometry (not shown), indicating that both compounds, 8-Fluo-cAMP and cAMP, addressed the same binding pocket in the surface-displayed HCN4 C-Linker-CNBD. It indicated as well that the flow cytometer-based quantification of 8-Fluo-cAMP binding could be a method to determine the binding of compounds addressing the CNBD of HCN4 in a quantitative manner. To support this hypothesis, the mean fluorescence intensity for each cAMP concentration (mFI_cAMP_) was normalized by the mFI of 50 nM 8-Fluo-cAMP (mFI_8-Fluo-cAMP_) and plotted against the cAMP concentration. This resulted in a curve indicating the displacement of 8-Fluo-cAMP by cAMP (Figure 3A). Half of the maximum displacement was obtained at a concentration of 13 ± 2 µM, representing the IC_50_ value of cAMP for 8-Fluo-cAMP binding to surface-displayed CNBD (Figure 3A). 

To evaluate the suitability of the assay in terms of ligand displacement studies, a second compound, 7-CH-cAMP (Figure S3A), was subjected to the same experiments. The concentration of 8-Fluo-cAMP was fixed at 50 nM as before, and different concentrations of 7-CH-cAMP ranging from 1 nM to 25 µM were applied. Again, the mFI value as obtained for each 7-CH-cAMP concentration (mFI_7-CH-cAMP_) was normalized by that of 8-Fluo-cAMP (mFI_8-Fluo-cAMP_) and plotted against the 7-CH-cAMP concentration. This resulted again in a displacement-binding curve (Figure S3B). An IC_50_ value for 7-CH-cAMP was determined to be 230 ± 41 nM and an inhibitory constant (K_i_) was calculated as 159 ± 29 nM. This K_i_-value determined for 7-CH-cAMP is about fivefold higher than the previously described K_d_-value of 30 nM for 7-CH-cAMP, which was determined by ITC measurements with HCN4 MBP-C-Linker-CNBD fusion protein [35]. The difference could be due to the different assays applied but it may as well be the results of two different proteins used in the different assays. 

### 2.5. Further Analysis of the Competitive Binding between 8-Fluo-cAMP and cAMP

To further confirm the competitive binding mechanism between cAMP and 8-Fluo-cAMP, cells displaying the CNBD were incubated with four different but fixed 8-Fluo-cAMP concentrations (50 nM, 150 nM, 250 nM, 500 nM) in the presence of increasing cAMP concentrations ranging from 100 nM to 1 mM, followed by flow cytometry and data analysis as described above. For each fixed concentration of 8-Fluo-cAMP, a typical displacement curve was obtained (Figure 3B). From the four different binding curves, an IC_50_ value for cAMP could be determined and turned out to be 13 ± 2 µM/16 ± 3 µM/23 ± 1 µM/27 ± 1 µM at 50 nM/150 nM/250 nM/500 nM 8-Fluo-cAMP, respectively. In the case of a competitive binding mode, increasing IC_50_ values of cAMP at higher 8-Fluo-cAMP concentrations were expected because higher competitor concentrations are necessary to reach a half-maximal displacement. Subsequently, the IC_50_ values obtained for cAMP were plotted against the concentration of 8-Fluo-cAMP. This resulted in a linear relationship, confirming the competitive binding mode between the two compounds (Figure 3C). Furthermore, the K_i_-value for cAMP could be graphically determined as the *y*-intercept is supposed to be equal to the K_i_ of cAMP [52]. A K_i_ value of 11.7 µM as determined here graphically was in good agreement with a calculated K_i_ value of 8.5 ± 2 µM obtained by the competition experiments. Both values are in a similar range as the K_d_ values determined before for cAMP in other studies, ranging from 0.8 µM (determined by ITC) [9] and 1.5 µM (SPR) [36] to 1–9 µM in Saturation Transfer Difference (STD)-NMR experiments [53]. In these studies, the direct binding affinity (K_d_) of cAMP to purified HCN4 C-Linker-CNBD was measured. In competitive binding studies, the binding affinity of the competitor is determined indirectly by fluorescently labeled ligand displacement. The calculated inhibitory constant K_i_ represents the dissociation constant of the inhibitor [54]. The obtained K_i_ values can be compared to the described K_d_ values because both represent the binding affinity of the compound. However, the way to determine binding affinities is different in both scenarios.

### 2.6. Investigating Ivabradine and Gabapentin in the C-Linker-CNBD Binding Assay

**Figure 4:**
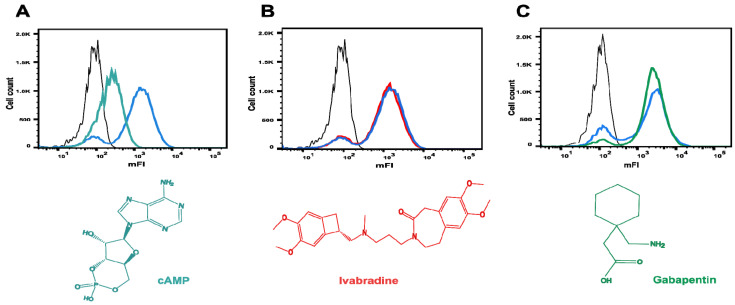
Flow cytometry histograms of the competitive binding assay with 50 nM 8-Fluo-cAMP and 100 µM competitor. *E. coli* BL21 cells without plasmid served as negative control (black). mFI of cells presenting the HCN4 C-Linker-CNBD after 8-Fluo-cAMP treatment are represented in blue. mFI measured after competitor addition are shown for (**A**) cAMP (aqua), (**B**) ivabradine (red) and (**C**) gabapentin (green).

The effect of ivabradine and gabapentin on 8-Fluo-cAMP binding was investigated to find out whether these two approved drugs could have an influence on the affinity of 8-Fluo-cAMP to CNBD. The binding assay was performed with 50 nM 8-Fluo-cAMP and 100 µM either ivabradine or gabapentin. *E. coli* BL21 cells without plasmid were treated identically and served as a control. In a similar experiment, incubation with 50 nM 8-Fluo-cAMP together with 100 µM cAMP served as an additional control. As expected, the incubation with cAMP resulted in a decreased mFI of cells displaying the CNBD in comparison with the same cells incubated with 8-Fluo-cAMP alone (Figure 4A). Ivabradine, an approved HCN isoform unselective pore channel blocker, was chosen for the analysis, because it is known to block the HCN channels by interacting from the intracellular site with a cavity formed below the pore, and hence, no impact on 8-Fluo-cAMP binding should occur [55]. As expected, there was no influence of 100 µM ivabradine on the binding of 50 nM 8-Fluo-cAMP to the CNBD (Figure 4B). Gabapentin is a drug with a wide range of indications including epilepsy, neuropathic pain disorder and off-label use for the treatment of bipolar disorders or anxieties [56]. Tae et al. showed the selective modulation of HCN4 voltage dependence by gabapentin; however, the mode of action remained unveiled [57]. Therefore, in this study, the effect of gabapentin on 8-Fluo-cAMP binding to the CNBD of HCN4 was tested. The flow cytometer analysis showed a slight increase in the population of cells displaying the HCN4 C-Linker-CNBD binding 8-Fluo-cAMP and a similar decrease in the population of cells not binding 8-Fluo-cAMP in the same experiment (Figure 4C). This could be due to an experimental variability of 8-Fluo-cAMP binding to the CNBD. In this experiment, 100 µM gabapentin had no impact on the binding of 50 nM 8-Fluo-cAMP to the CNBD. This is the first indication that gabapentin is not interacting with the CNBD of HCN4 channels; however, further investigations are required to identify its mode of action with HCN4 channels. 

### 2.7. Screening for Inhibitors

In total, 76 compounds from an in-house library were analyzed on 8-Fluo-cAMP displacement. The assay conditions were identical to those described for ivabradine and gabapentin. The selection from the in-house library contained compounds with different chemical scaffolds including benzimidazole derivatives [58] (Table S1), indenoindole and indenoindoledione derivatives [59,60,61,62] (Table S2), phenoxazine and phenothiazine derivatives [63,64] (Table S3), acridine derivatives [65] (Table S4), naphthofuran and naphthothiophene derivatives [66,67,68] (Table S5). Unfortunately, none of these compounds had an effect of more than 10% on the mFI values obtained with 8-Fluo-cAMP and hence, no novel HCN4 C-linker CNBD ligand could be identified.

## 3. Discussion

A flow cytometry-based assay with surface-displayed HCN4 C-Linker-CNBD was established as a method for the investigation of ligand binding to the CNBD. Compared to other methods described before, there is neither a need for protein purification nor the need for enhancing the stability and solubility of the C-linker-CNBD by an additional protein domain, such as MBP [35,39]. Protein stability appeared to be no issue when fused to the autotransporter translocator domains. As no protein purification is required, it appears that the costs of the assay as described are lower than those of other assay applied for the same purpose. In addition, it seems quite convenient because only bacterial cells, simple to cultivate, were incubated with a commercially available fluorescent ligand (8-Fluo-cAMP) and the compound which shall be analyzed on HCN4 C-linker-CNBD binding, with a fluorescence readout at the end. The binding constants determined for 8-Fluo-cAMP, 7-CH-cAMP and cAMP are in agreement with those determined before by other means [9,35,47,53]. 

In the current setup of the assay, around 35 compounds can be analyzed per day when samples are manually prepared. The throughput could be increased by performing the assay in, e.g., 96-well plates instead of reaction tubes as applied here and through the use of robotics. For flow cytometry, 50,000 events per sample were measured in approximately 30–45 s, which means that the throughput of the measurement was around 80–120 samples per hour.

The assay as described here could be used to screen compound libraries on new binders to the CNBD of HCN4, which then could be analyzed in further experiments, e.g., patch clamp on channel modulation. Furthermore, drugs already known to modulate HCN channels with an unclear mode of action can be specified as having a potential effect on the CNBD. 

A clear limitation of the assay is that it can exclusively differentiate between binding and non-binding compounds. As only the intracellular ion channel part is expressed on the cell surface, it is not possible to distinguish between compounds inhibiting or modulating the cAMP-induced ion channel effects. Therefore, compounds identified by the assay must be tested in whole-cell experiments to investigate their impact on ion channel voltage-dependent properties. However, it appears that the assay as described can be helpful as a prescreening method before more expensive and time-consuming eukaryotic cell experiments are conducted. It has been described before that proteins expressed as monomers using autodisplay can form dimeric [69,70] or tetrameric [50,71] structures at the cell surface. This is supposed to be due to the mobility of the anchoring β-barrel domain within the outer membrane after transport [41] and the affinity of the protein subunits to each other as displayed. The C-Linker-CNBD of HCN4 is supposed to form tetramers. However, in our expression studies, we could not detect any hint of tetramerization. Therefore, for the time being, we assume the C-linker CNBD of HCN4 to be monomeric at the cell surface.

In a further embodiment, surface displays of the CNBDs of the other HCN channel subtypes 1, 2 and 3 could be performed and applied for compound selectivity testing. Moreover, mutational analysis of the binding pocket in the CNBD could be performed by standard methods to investigate the importance of different amino acids on ligand binding, which could serve as a basis for the design of subtype-specific compounds. These features of the assay described here could contribute to drug discovery approaches targeting the HCN CNBD and reveal new insights into the function of HCN channels. Overall, its robustness, the comparably low costs, a considerable throughput and the simple implementation of the assay make it a convenient screening method for HCN4 C-linker CNBD ligands.

## 4. Materials and Methods

### 4.1. Chemicals and Materials

7-Deazaadenosine-3,5′-cyclic monophosphate (7-CH-cAMP) sodium salt and 8-(2-[Fluoresceinyl]aminoethylthio)adenosine-3′,5′-cyclic monophosphate (8-[Fluo]-cAMP) sodium salt were obtained from Biolog (Bremen, Germany). 3-[(3-Cholamidopropyl)-dimethylammonio]-1-propansulfonate hydrate (CHAPS), Adenosine-3′,5′-cyclic monophosphate (cAMP), Phenylmethylsulfonylfluorid (PMSF) and NaCl were obtained from Sigma-Aldrich (Schnelldrof, Germany). Kanamycin sulfate, L-arabinose, Proteinase K and standard media ingredients were purchased from Carl Roth (Karlsruhe, Germany). Dipotassiumhydrogenphosphate (K_2_HPO_4_) was sourced from Merck (Darmstadt, Germany); Disodiumhydrogenphosphate (Na_2_HPO_4_) was obtained from VWR Chemicals (Darmstadt, Germany) and KCl from Applichem (Darmstadt, Germany). Myc Tag monoclonal mouse IgG1 antibody and the Goat anti-Mouse IgG (H + L) Secondary Antibody and DyLightTM 633 were obtained from Thermo Fisher Scientific (Braunschweig, Germany). The DNA string encoding the Maltose-binding-protein (MBP)-hHCN4-C-Linker-CNBD optimized for *E. coli* was synthesized by Invitrogen Thermo Fisher Scientific/Life Technologies GmbH (Darmstadt, Germany). Gabapentin was purchased from TCI (Zwijndrecht, Belgium). Ivabradine was provided by Prof. Seebohm (Institute for Genetics of Heart Diseases, IfGH), Department of Cardiovascular Medicine, University Hospital Münster, 48149 Münster, Germany).

### 4.2. Bacterial Strains and Plasmid Construction

Outer membrane protease (OmpT)-deficient *E. coli* BL21 [72,73] strain was used for the surface expression of HCN4 C-Linker-CNBD. To prepare the passenger domain, the synthesized DNA string encoding the MBP-HCN4-C-Linker-CNBD served as a template for PCR amplification of the HCN4 C-Linker-CNBD only. The corresponding primers used (5′-AAA ACT CGA GGA TAG CAG CCG TCG TCA GTA TC-3′ and 5′-TTT TGG TAC CAT GCA GCA GAA TAC-3′) were flanked with terminal XhoI and KpnI restriction sites. The amplified PCR product was inserted into the pDG01 [44] expression vector via restriction and ligation. The resulting plasmid pMJ03 carried the autotransporter fusion gene for the surface display of HCN4 C-Linker-CNBD containing a N-terminal His-Tag and C-terminal Myc-Tag. Using the primer 5′-GAG AAT CTT TAT TTT CAG GGC CTG ACC AAC AAT GGC ACG CTG ATG-3′ and 5′-GCC CTG AAA ATA AAG ATT CTC CAG ATC CTC TTC TGA GAT GAG TTT TTG TTC-3′, an additional Tobacco Etch Virus (TEV) cleavage site was introduced at the C-terminal site of the passenger protein, creating the plasmid pMJ13. As this led to an enhanced ligand-binding signal (data not shown), the work was continued with this plasmid. As a negative control, the plasmid pMJ22 was constructed encoding MBP for surface display. The MBP encoding sequence was amplified from the template DNA-string using the primer 5′-AAA ACT CGA GAA AAT CGA AGA GGG-3′ and 5′-TTT TGG TAC CGC TGC TGC TAT TGG TCT G-3′. The amplified DNA was inserted into the pMJ13 backbone via restriction and ligation using the XhoI/KpnI sites, hence replacing the HCN4-C-Linker-CNBD by the MBP encoding sequence.

### 4.3. Culture Conditions and Sample Preparation 

Bacteria were cultivated in lysogeny broth (LB) medium (5 g/L yeast extract, 10 g/L peptone, 10 g/L NaCl) supplemented with 50 µg/mL kanamycin. An overnight culture was grown, followed by 1:100 inoculation into fresh LB medium. Cells were incubated at 37 °C (200 rpm) until an OD578 of 0.5–0.6 was reached. Gene expression was induced by the addition of 0.2% final concentration of L-arabinose for 2 h at 23 °C (200 rpm). Cells were harvested by centrifugation (3850× *g*, 4 °C, 5 min) and stored in 1 mL PBS (2.7 mM KCl, 137 mM NaCl, 2 mM KH_2_PO_4_, 10 mM Na_2_HPO_4_, pH 7.4) overnight at 4 °C.

### 4.4. Proteinase K Digestion

After culturing, cells were harvested by centrifugation (3850× *g*, 4 °C, 5 min) and suspended in 1 mL PBS. A total of 12.5 µL of Proteinase K (5 mg/mL) was added to the sample and it was incubated for 1 h at 37 °C (200 rpm). After the addition of 5 mM PMSF, cells were harvested and stored in 1 mL PBS overnight at 4 °C.

### 4.5. Immunolabeling

Bacterial cells were suspended and washed three times by sedimentation and suspension in 5 mL ice-cold sterile PBS (3850× *g*, 4 °C, 5 min). The sediment was suspended in 5 mL PBS and an OD_578_ of 0.35 was adjusted for each sample followed by centrifugation (13,000× *g*, 4 °C, 1 min). Cells were suspended in 100 µL PBS followed by the addition of 1 µL Myc-Tag monoclonal mouse IgG1 antibody. After incubation for 1 h at RT and 600 rpm, cells were harvested and washed three times by sedimentation and suspension with 500 µL PBS (13,000× *g*, 4 °C, 1 min). Cells were suspended in 100 µL PBS and 2 µL Goat anti-Mouse IgG (H + L) Secondary Antibody, DyLight^TM^ 633 were added to the samples and incubated for 1 h at RT and 600 rpm. After the incubation, the cells were harvested by centrifugation and washed three times as described above. Cells were suspended in 200 µL PBS and analyzed by flow cytometry. 

### 4.6. Ligand-Binding Assays

For ligand-binding studies, cells were adjusted to an OD578 of 0.35 followed by centrifugation (12,000× *g*, 4 °C, 2 min). The sediment was suspended in ice-cold sterile PBS containing 0.1% CHAPS. Corresponding volumes of 8-Fluo-cAMP were added to the samples to create a concentration series reaching from 1 nM to 4 µM. The final sample volume was 100 µL. Cells were incubated for 30 min at 30 °C with vigorous shaking (600 rpm). After harvesting by centrifugation, 100 µL ice-cold sterile PBS was added, followed by flow cytometry analysis. For the competitive binding assay, cells were preincubated with 50nM 8-Fluo-cAMP. After 10 min, the competitor was added and incubated to a final incubation time of 30 min at 30 °C (600 rpm). This was followed by the steps described previously. The concentration for cAMP ranged from 100 nM to 1 mM and for 7-CH-cAMP—from 1 nM to 25 µM. Ivabradine and gabapentin were tested at 100 µM. 

### 4.7. Flow Cytometry Analysis 

A total of 50,000 cells per sample were analyzed with a FACS Aria III flow cytometer (BD Biosciences, Franklin Lakes, NJ, USA). For immunolabeling, an excitation wavelength of 633 nm and an emission wavelength filter of 660/20 nm were used. Ligand-binding analysis was performed using an excitation wavelength of 488 nm and an emission wavelength filter of 530/30 nm.

### 4.8. Data Analysis

For data analysis, the GraphPad Prism software version 5.02 was used. For ligand-binding studies, the mean fluorescence intensity (mFI) of each sample was normalized to the mean maximum mFI (mFI_max_) obtained for 4 µM 8-Fluo-cAMP as the highest concentration used. The resulting value corresponding to the ligand fraction bound was plotted against the ligand concentration. The K_d_-value for 8-Fluo-cAMP was determined using Equation (1) following Hunter and Cochran (2016) [48].

(1) $$y=\frac{x}{\left(K_{d}+x\right)}$$

For competitive binding studies, the mFI values resulting for 50 nM 8-Fluo-cAMP with competitor added (mFI_competitor_) were normalized to mFI of 50 nM 8-Fluo-cAMP only (mFI_8-Fluo-cAMP_). IC_50_ values were determined using the GraphPad Prism equation Binding-Competitive-One Site-Fit logIC_50_ (2).

(2) $$y=y_{min}+(y_{max}-y_{min})/(1+10^{x-log{IC}_{50}})$$

The Binding-Competitive-One Site-Fit Ki Equation (3) was used to determine the competitor Ki values.

(3) $$log{IC}_{50}=\log\left(10^{log\mathrm{Ki}\times \left(1+\frac{c\left(ligandnM\right)}{K_{d}\left(ligandnM\right)}\right)}\right)$$

The histogram plots were illustrated using the software FlowJo 10 (LLC, Ashland, OP, USA). Each histogram plot was recorded in three technical replicates for a single biological replicate. For each concentration, a total of three biological triplicates were analyzed accordingly. 

## Data Availability

Data is contained within the article and supplementary material.

## Supplementary Materials

The following supporting information can be downloaded at: https://www.mdpi.com/article/10.3390/ph16050710/s1, Figure S1: Proof of HCN4 C-Linker-CNBD surface display via immunolabeling and flow cytometry, Figure S2: Analysis of equilibrium conditions and ligand depletion, Figure S3: Structure of 7-CH-cAMP, competitive binding assay with 8-Fluo-cAMP and 7-CH-cAMP, Table S1: benzimidazole derivatives; Table S2: indenoindole and indenoindoledione derivatives; Table S3: phenoxazine and phenothiazine derivatives; Table S4: acridine derivatives; Table S5: naphthofuran and naphthothiophene derivatives.
