# Supplementary material for: A Novel Flow Cytometry-Based Assay for the Identification of HCN4 CNBD Ligands
Source: Pharmaceuticals (Basel). 2023 May 7;16(5):710. doi: 10.3390/ph16050710 (PMC10224427; doi:10.3390/ph16050710)
Supplement: Supplementary file 1 [file pharmaceuticals-16-00710-s001.zip › pharmaceuticals-2342707-supplementary.pdf]

Supporting Information for

A Novel Flow Cytometry-Based Assay for the Identification of HCN4 CNBD Ligands

Magdalena N. Wojciechowski, Sebastian Schreiber and Joachim Jose *

University of Münster, Institute of Pharmaceutical and Medicinal Chemistry, Pharmacampus, 48149 Münster, Germany
*   Correspondence: joachim.jose@uni-muenster.de

Table of contents:

Figure S1: Prove of HCN4 C-Linker-CNBD surface display *via* immunolabeling and flow cytometry.

Figure S2: Analysis of equilibrium conditions and ligand depletion

Figure S3: Structure of 7-CH-cAMP, competitive binding assay with 8-Fluo-cAMP and 7-CH-cAMP.

Table S1: Benzimidazole-derivatives tested for 8-Fluo-cAMP displacement

Table S2: Indenoindole- and indenoindoledione- derivatives tested for 8-Fluo-cAMP displacement

Table S3: Phenoxazine- and Phenothiazine- derivatives tested for 8-Fluo-cAMP displacement

Table S4: Acridine- derivatives tested for 8-Fluo-cAMP displacement

Table S5: Naphthofurane- and Naphthothiophene- derivatives tested for 8-Fluo-cAMP displacement

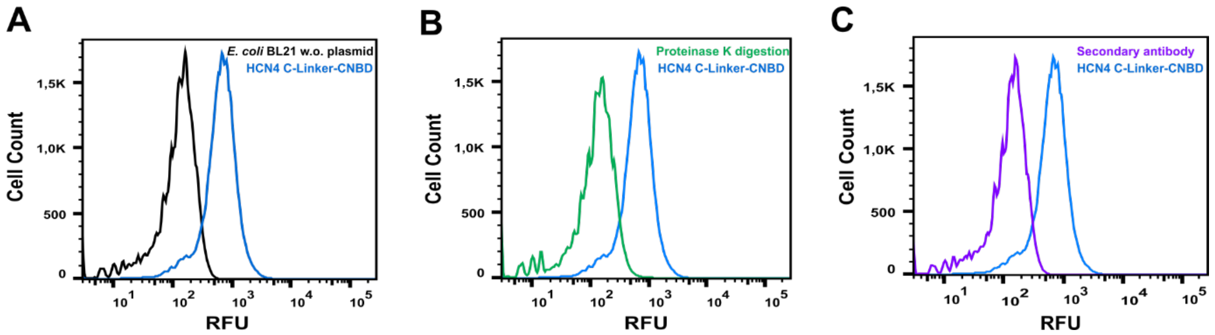

Figure S1. Prove of HCN4 C-Linker-CNBD surface display *via* immunolabeling and flow cytometry. (**A**) Histogram of *E. coli* BL21 cell without plasmid (black) and *E. coli* BL21 cells displaying the HCN4 C-Linker-CNBD (blue) treated with primary anti Myc-tag antibody and secondary antibody conjugated to the DyLight™633 fluorophore. (**B**) Immunolabeling histogram of cells displaying the HCN4 C-Linker-CNBD before (blue) and after Proteinase K digestion (green). (**C**) Histogram of cells presenting the HCN4 C-Linker-CNBD labelled with secondary antibody only (purple) or primary and secondary antibody (blue).

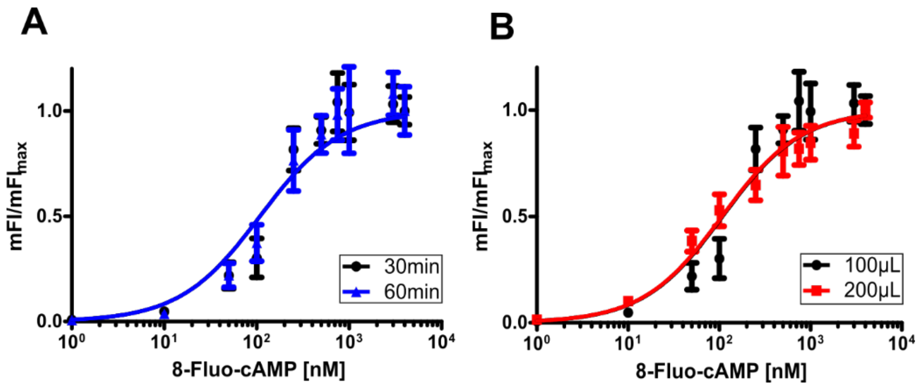

Figure S2. Analysis of equilibrium conditions and ligand depletion. (**A**) Comparison of the ligand binding curves obtained after 30min (black) and 60min (blue) incubation time. (**B**) Comparison of the ligand binding curves after variation of the sample volume 100µL (black) and 200µL (red).

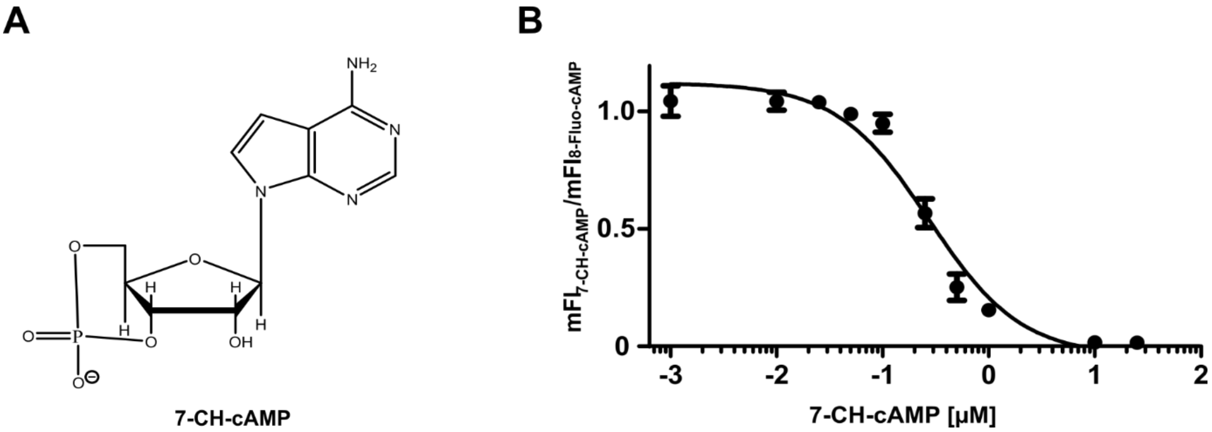

Figure S3. Competitive binding assay with 8-Fluo-cAMP and 7-CH-cAMP. (**A**) Structure of 7-CH-cAMP. (**B**) Displacement binding curve obtained for 7-CH-cAMP ranging from 1nM-25μM and a fixed concentration of 8-Fluo-cAMP at 50nM. Mean fluorescence measured for each 7-CH-cAMP concentration ($mFI_{7\text{-CH-cAMP}}$) was normalized to mean fluorescence for 50nM 8-Fluo-cAMP only ($mFI_{8\text{-Fluo-cAMP}}$) and plotted against competitor concentration.

Table S1. Benzimidazole-derivatives tested for 8-Fluo-cAMP displacement

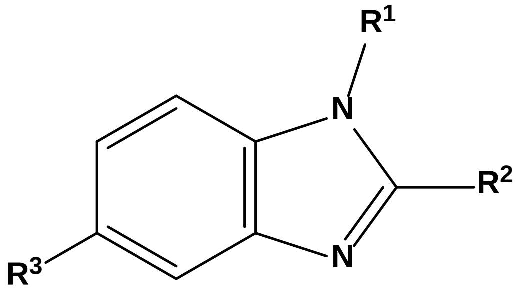

Name	R^1	R^2	R^3
1	H	CH_2-(*p*-OMe)Ph	H
2	H	(*p*-Cl)Ph	CH_2-OH
3	H	Ph	CH_2-OH
4	Ph	(2,6-Cl)Ph	H
5	H	(*p*-Me)Ph	CH_2-OH
6	H	(*p*-N(CH3)2Ph	CH_2-OH

Table S2. Indenoindole- derivatives tested for 8-Fluo-cAMP displacement

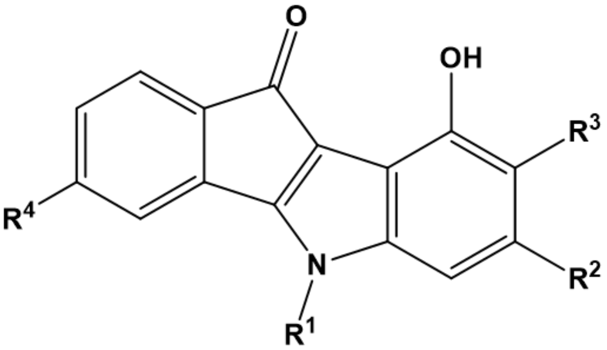

Name	R1	R2	R3	R4
7	$CH(Me)_2$	2-furan	H	H
8	$CH(Me)_2$	H	H	H
9	CH_2-Ph	H	H	H
10	$CH(Me)_2$	COOMe	H	H
11	$CH(Me)_2$	COOH	H	H
12	$CH(Me)_2$	Me	Me	H
13	$CH(Me)_2$	COOEt	Me	H
14	CH_2-CH_2-Br	H	H	H
15	CH_2-CH_2-2-(5-OMe)indole	H	H	H
16	$CH(Me)_2$	Me	H	OCH_2-CH=C$(Me)_2$
17	CH_2-CH_2-(*o*-OMe)Ph	Me	H	H
18	H	H	H	H

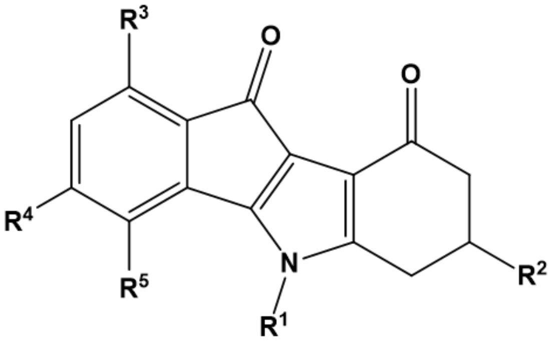

Name	R1	R2	R3	R4	R5	R6
19	$CH(Me)_2$	COOH	H	H	H	H
20	$CH(Me)_2$	COOH	Me	H	H	H
21	$CH(Me)_2$	Me	H	OMe	H	H
22	$CH_2CH(Me)_2$	H	H	H	H	H
23	CH_2CH_2OMe	H	H	H	H	H
24	$CH_2CH_2N(CH_2CH_3)_2$	H	H	H	H	H
25	$CH(Me)_2$	Me	H	OH	H	H
26	$CH(Me)_2$	Me	H	H	H	OH
27	$CH(Me)_2$	Me	H	H	H	OCH_2CH=C$(Me)_2$
28	$CH(Me)_2$	CH_2CH_3	H	OH	H	H
29	$CH(Me)_2$	CH_2CH_3	H	H	H	OH
30	$CH(Me)_2$	CH_2CH_3	H	H	H	OCH_2CH=C$(Me)_2$

31	CH(Me)$_2$	H	H	H	OCH2COOH	H
32	CH(Me)$_2$	H	H	H	OCH2CH2CH2 COOH	H
33	CH$_2$CH$_2$-(*o*-OMe)Ph	Me	H	OCH$_2$CH=C(Me)$_2$	H	H
34	CH$_2$CH$_2$(*o*-OMe)Ph	Me	H	H	H	OCH$_2$CH=C(Me)$_2$
35	CH$_2$CH$_2$Ph	Me	H	H	H	OCH$_2$CH=C(Me)$_2$
36	H	H	H	H	H	H
37	Me	H	H	H	H	H
38	CH$_2$CH$_3$	H	H	H	H	H

Table S3. Phenoxazine- and Phenothiazine-derivatives tested for 8-Fluo-cAMP displacement

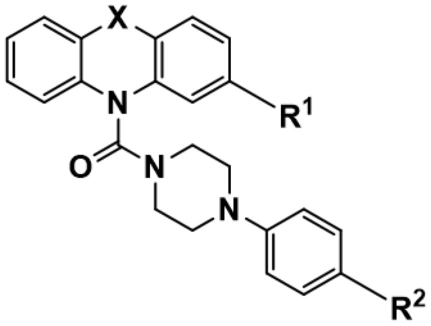

Name	X	R^1	R^2
39	O	H	H
40	O	H	Me
41	O	H	CF$_3$
42	O	H	NO$_2$
43	O	H	CN
44	S	Cl	OMe
45	S	H	OMe
46	S	H	CN

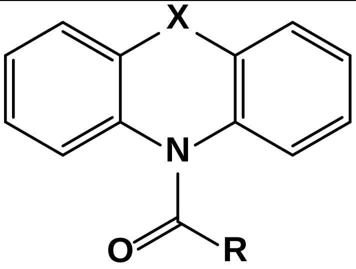

Name	X	R
47	O	NH-CH$_2$(*p*-OMe)Ph
48	O	NH-CH$_2$(*m*-OMe)Ph
49	O	1-imidazole-4(*m*-OMe)Ph
50	S	1-imidazole-4(*m*-OMe)Ph

Table S4. Acridine- derivatives tested for 8-Fluo-cAMP displacement

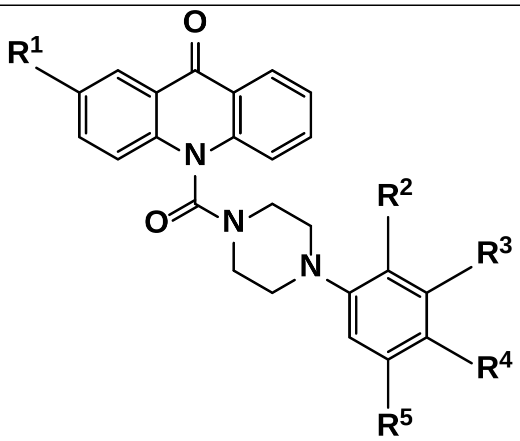

Name	R1	R2	R3	R4	R5
51	H	H	H	OMe	H
52	H	H	H	H	H
53	H	H	H	Cl	H
54	OMe	OMe	H	H	H
55	H	H	OMe	OMe	OMe
56	H	OMe	H	OMe	H
57	H	H	H	Cl	H

Table S5. Naphthofuran- and Naphthothiophene- derivatives tested for 8-Fluo-cAMP displacement

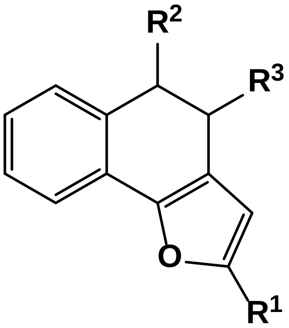

Name	R1	R2 = R3
58	CH_2CH_3	=O
59	(*p*-OMe)Ph	=O
60	3-pyridine	=O
61	OMe	=O
62	$N(CH_2CH_3)_2$	=O
62	$N(CH(Me)_2)_2$	OMe
64	$NHCH_2CH_2OH$	OMe

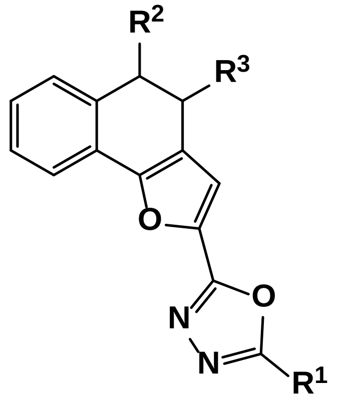

Name	R^1	$R^2 = R^3$
65	CH_2CH_3	OMe
66	CH_2CH_3	=O
67	Ph	OMe
68	Ph	=0

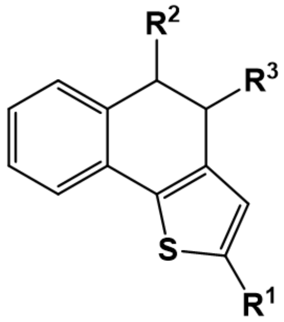

Name	R^1	$R^2 = R^3$
69	2-thiophene	=O
70	OMe	=O
71	OCH_2CH_3	=O
72	OPh	=O
73	$N(CH(Me)_2)_2$	OMe
74	COOH	OMe
75	COOH	=O
76	$NHCH_2CH_2OH$	OMe
